# Supplementary material for: Splice-Junction-Based Mapping of Alternative Isoforms in the Human Proteome
Source: Cell Rep. Author manuscript; Available in PMC 2020 Jan 15. (PMC6961840; doi:10.1016/j.celrep.2019.11.026)
Supplement: 3 [file NIHMS1546469-supplement-3.zip › DF2/PXD009737/TestisTrypsin-32-Q9GZY8-NDSLYGISNIDTTIEGTSDDLTVVDAASLR.pdf]

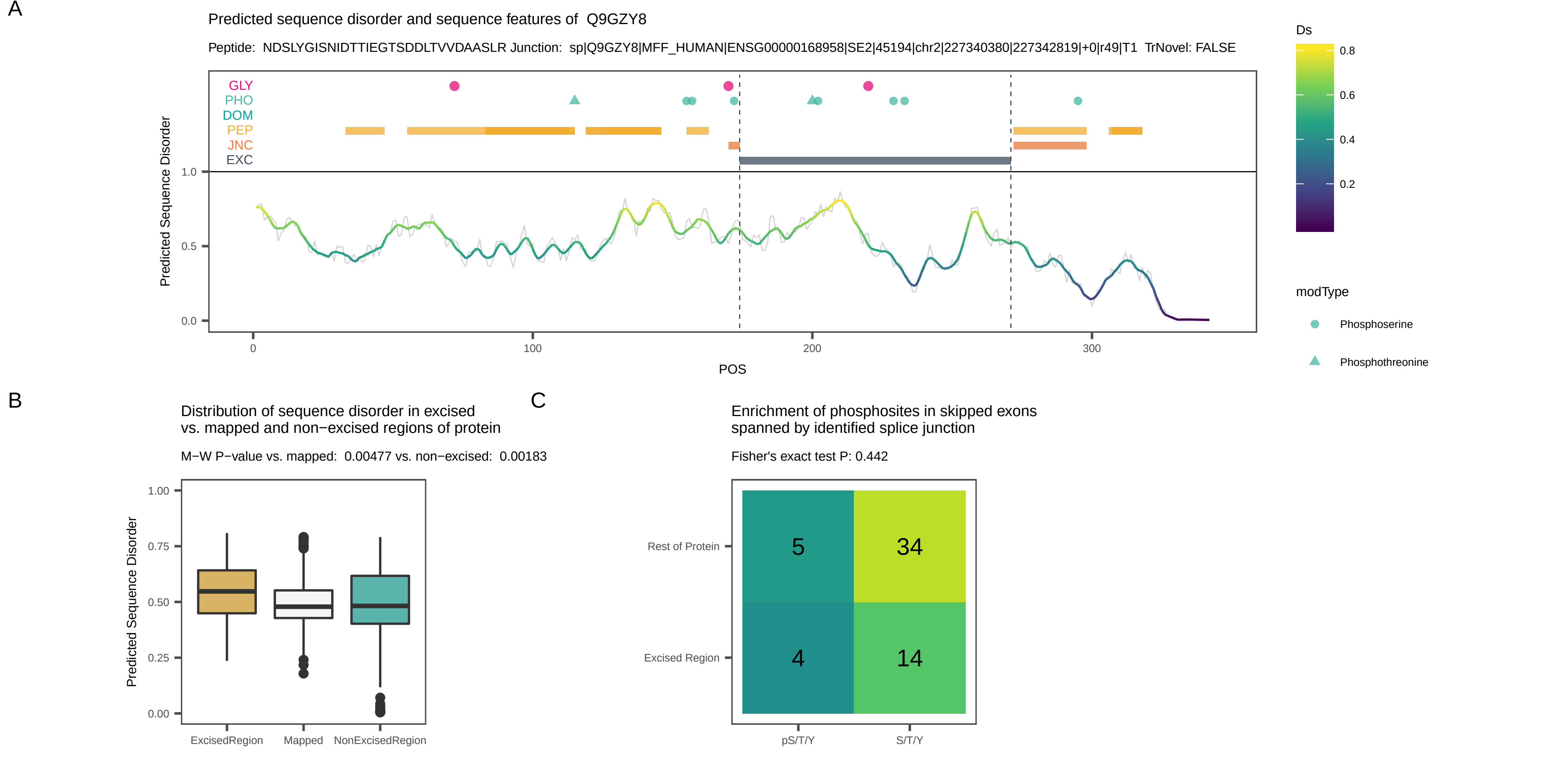
A
Predicted sequence disorder and sequence features of Q9GZY8
Peptide: NDSLYGISNIDTTIEGTSDDLTVVDAASLR Junction: sp|Q9GZY8|MFF_HUMAN|ENSG00000168958|SE2|45194|chr2|227340380|227342819|+0|r49|T1 TrNovel: FALSE
GLY
PHO
DOM
PEP
JNC
EXC
Predicted Sequence Disorder
POS
Ds
modType
Phosphoserine
Phosphothreonine
B
Distribution of sequence disorder in excised vs. mapped and non−excised regions of protein
M−W P−value vs. mapped: 0.00477 vs. non−excised: 0.00183
Predicted Sequence Disorder
ExcisedRegion
Mapped
NonExcisedRegion
C
Enrichment of phosphosites in skipped exons spanned by identified splice junction
Fisher's exact test P: 0.442
Rest of Protein
Excised Region
pS/T/Y
S/T/Y
5
34
4
14
